# A Systematic Review investigating the Effectiveness of Exercise training in Glycogen Storage Diseases

**DOI:** 10.1177/26330040221076497

**Published:** 2022-02-23

**Authors:** Claire Bordoli, Elaine Murphy, Ian Varley, Graham Sharpe, Philip Hennis

**Affiliations:** Sport, Health and Performance Enhancement (SHAPE) Research Centre, Nottingham Trent University, Clifton Lane, Clifton, Nottingham NG11 8NS, UK; Charles Dent Metabolic Unit, The National Hospital for Neurology and Neurosurgery, London, UK; Sport, Health and Performance Enhancement (SHAPE) Research Centre, Nottingham Trent University, Nottingham, UK; Sport, Health and Performance Enhancement (SHAPE) Research Centre, Nottingham Trent University, Nottingham, UK; Sport, Health and Performance Enhancement (SHAPE) Research Centre, Nottingham Trent University, Nottingham, UK

**Keywords:** endurance training, exercise, glycogen storage disease, respiratory training, review, strength training

## Abstract

**Introduction:**

Glycogen storage diseases (GSDs) are rare inborn errors of carbohydrate metabolism typically with skeletal muscle and liver involvement. In those with skeletal muscle involvement, the majority display symptoms of exercise intolerance which can cause profound exercise limitation and impair everyday living and quality of life (QoL). There are no curative treatments for GSDs, thus therapeutic options, such as exercise training, are aimed at improving QoL by alleviating signs and symptoms. In order to investigate the effectiveness of exercise training in adults with GSDs, we systematically reviewed the literature.

**Methods:**

In this review we conducted searches within SCOPUS and MEDLINE to identify potential papers for inclusion. These papers were independently assessed for inclusion and quality by two authors. We identified 23 studies which included aerobic training, strength training or respiratory muscle training in patients with McArdles (*n* = 41) and Pompe disease (*n* = 139).

**Results:**

In McArdle disease, aerobic exercise training improved aerobic capacity (VO_2_ peak) by 14–111% with further benefits to functional capacity and well-being. Meanwhile, strength training increased muscle peak power by 100–151% and reduced disease severity. In Pompe disease, a combination of aerobic and strength training improved VO_2_ peak by 9–10%, muscle peak power by 64%, functional capacity and well-being. Furthermore, respiratory muscle training (RMT) improved respiratory muscular strength [maximum inspiratory pressure (MIP) increased by up to 65% and maximum expiratory pressure (MEP) by up to 70%], with additional benefits shown in aerobic capacity, functional capacity and well-being.

**Conclusion:**

This adds to the growing body of evidence which suggests that supervised exercise training is safe and effective in improving aerobic capacity and muscle function in adults with McArdle or Pompe disease. However, the literature base is limited in quality and quantity with a dearth of literature regarding exercise training in other GSD subtypes.

## Plain Language Summary

A Systematic Review investigating the Effectiveness of Exercise training in Glycogen Storage Diseases

**Background**: Glycogen storage diseases (GSDs) are a group of rare inherited metabolic disorders typically affecting carbohydrate metabolism within the skeletal muscle and liver. In those where the skeletal muscle is affected, the majority display exercise intolerance which can impact everyday living and quality of life. No curative treatments for GSDs exist, therefore exercise training offers a therapeutic option to reduce symptoms and enhance patient’s quality of life. In order to investigate the effectiveness of exercise training as a therapeutic option, we systematically reviewed the literature.

**Study Characteristics:** We found 23 studies which included aerobic training, strength training and respiratory muscle training in adults with certain types of GSD including McArdles (n = 41) and Pompe disease (n = 139).

**Key Results:**

- In McArdle disease, aerobic exercise improved aerobic performance, with further benefits to functional capacity and well-being. Meanwhile, strength training increased muscular strength and reduced disease severity.
- In Pompe disease a combination of aerobic and strength training improved aerobic capacity, muscular strength, functional capacity and well-being. Furthermore, respiratory muscle training (RMT) improved the strength of respiratory muscles with further benefits in aerobic capacity, functional capacity and well-being.

**Implications:** This systematic review indicates that supervised exercise training is safe and effective in improving aerobic capacity and muscle function in adults with McArdle or Pompe disease. However, the effectiveness of exercise training in other GSDs is as yet unknown.

## Introduction

Glycogen storage diseases (GSDs) are a rare heterogeneous group of inherited disorders of metabolism (IEM) usually caused by single pathogenic variants in genes that encode enzymes involved in carbohydrate metabolism.^1^ This results in enzyme deficiencies and subsequent defects of glycogen degradation, glycolysis or paradoxically glycogen synthesis.^2^ Typically, symptoms manifest in skeletal muscle and liver,^3,4^ but the kidneys, heart and in some cases the brain can also be affected.^5,6^ The specific enzyme deficiency and tissue involvement are used to classify people to one of at least 16 recognised GSD types,^7^ which currently range from Type 0 to Type XV.^8^ The genetic defects, clinical features and epidemiology of each GSD are summarised by Kanungo *et al.*^5^ GSDs manifest along a disease spectrum from being asymptomatic in some patients towards serious pathophysiological implications in others, which can impact peoples’ physical health, quality of life (QoL) and life expectancy.^5^

The physical manifestations of GSDs can become apparent from early childhood to late adulthood, with symptoms and the severity of symptoms varying greatly between different types of GSDs.^9–11^ They are broadly categorised into those with hepatic involvement (GSD0a, GSDIa, GSDIb, GSDVI, GSDIXA1, GSDIXA2, GSDIXc), those with skeletal muscle involvement (GSD0b, GSDII, GSDV, GSDVII, GSDIXD, GSDX, GSDXI, GSDXII, GSDXIII, GSDX1V, GSDXV) and those with both hepatic and skeletal muscle involvement (GSDIII, GSDIV, GSDIXb).^5^ Those with hepatic involvement commonly present with fasting hypoglycaemia, with or without hepatomegaly and liver disease.^12^ This can influence exercise tolerance due to the direct effects of liver glycogen content on exercise capacity as shown in rodents^13^ and the indirect effect of glycogen via its role in the maintenance of blood glucose homeostasis.^14^ In contrast where there is skeletal muscle involvement, skeletal myopathy is present.^12^ Those with skeletal muscle involvement can typically be divided into those showing static symptoms with loss of muscle mass and strength (GSDII, GSDIII) and those with dynamic exercise-related symptoms of fatigue, muscle pain and contractures, often associated with exercise-induced muscle damage (GSDV, GSDVII, GSDIXD, GSDX, GSDXIV).^15^ However, clinically these phenotypes can overlap, and precise classification can be challenging.^15^ In the GSDs with muscle involvement, exercise intolerance can lead to compromised habitual functioning, with increased morbidity and even premature death in some.^16–20^ In addition, as a likely consequence of exercise intolerance, many people with GSDs lead a sedentary lifestyle, which itself is associated with unwanted metabolic adaptations and further health issues.^21^

There are currently no curative treatments available for GSDs, thus therapeutic options are aimed at improving QoL by alleviating signs and symptoms.^22^ Dietary treatment varies based on the underlying enzyme defect and pathophysiology. Within hepatic GSDs nutritional therapy focusses upon preventing hypoglycaemia although there is a lack of general consensus on the optimal treatment.^7,8^ Enzyme replacement therapy (ERT) is an emerging drug treatment, which has proven benefits in Pompe disease (GSD II) but is not currently available for most GSD subtypes.^23^ Other supportive measures such as noninvasive ventilation (NIV) and cough assist devices are also employed for respiratory support and airway clearance in late-onset Pompe disease (LOPD) and GSDIII.^10,24^ Although the primary treatments of diet modification are beneficial for GSDs with hepatic and skeletal involvement (particularly in Types 0, I, III, VI, IX and XI) and ERT is relatively successful in reducing symptoms, impairments in functional capacity and QoL still persist.^7,8^ As such, there is a need to identify other treatments to accompany diet and ERT to further improve the health outcomes of people with GSDs. One such intervention is physical exercise training.

Exercise as an intervention may seem counterintuitive to many patients and clinicians given the severe exercise intolerance associated with many GSDs.^20^ However, increasingly, evidence suggests exercise can be beneficial in reducing symptoms and increasing QoL, rather than accelerating the disease.^15^ The three primary exercise interventions considered as treatments for GSDs are aerobic, resistance and respiratory muscle training.

Endurance exercise acts as a powerful inducer of metabolic changes in skeletal muscle. Chronic adaptations associated with training include improvements in substrate delivery to contracting muscles and an increased ability to oxidise non-esterified fatty acids^15^ at the same absolute and relative intensity post training.^25^ For this reason, in McArdle disease, improvements in aerobic capacity and work rate have been found, leading to greater exercise tolerance.^17,26,27^ Theoretically, endurance exercise could potentially have important benefits to those GSDs with hepatic involvement too, as this shift towards an increased reliance on fat as a fuel and the reduction in plasma glucose oxidation rates would subsequently be protective against hypoglycaemia. Meanwhile, strength training can reverse muscle weakness and atrophy, attenuating disease severity.^28^ Respiratory muscle training (RMT) is an emerging treatment in Pompe, involving resistance exercise specifically targeting the respiratory muscles, aiming to alleviate significant respiratory weakness.^29^ These exercise interventions will also combat the physical inactivity seen across the GSD spectrum and in doing so may improve overall general health, fitness and QoL.^30,31^

Researchers and clinicians have promoted the potential therapeutic benefits of exercise training for people with GSDs for many years. Supervised training programmes have even been included in consensus guidelines for those with Pompe disease.^32,33^ However, despite the beneficial effects of exercise training in GSDs being acknowledged, the research supporting the utility of exercise training in GSDs is sparse and heterogeneous, most likely due to the rarity of these diseases. To date, the only previous systematic review identified three studies and concluded that aerobic exercise effectively induces adaptations in cardiac, metabolic and skeletal muscle activity without adverse events in those with McArdle disease.^34^ However, no other GSDs were reviewed by Quinlivan *et al.*^34^ and since its publication a number of exercise intervention studies have been published, including a randomised controlled trial (RCT).^35^ As such, several questions remain, which include: Does current literature support the use of exercise training for people with GSD? Which GSD subtypes benefit from exercise training? Which training modalities are effective? And do patients adhere to prescribed exercise interventions? We aim to systematically review the current literature using a defined and reproducible strategy to investigate the broad impact of exercise training programmes across the GSD spectrum and establish the effects that various exercise interventions have on markers of cardiorespiratory and aerobic performance, muscular strength, functional capacity and well-being. In doing so, we aim to determine the feasibility and utility of using exercise training as a treatment option in those with GSDs to inform further research, clinical guidelines and practical recommendations.

## Methods

This systematic review is reported in line with the Preferred Reporting Items for Systematic Reviews and Meta-Analyses^36^ (Additional file 1).

### Eligibility

#### Criteria for inclusion of publications within this review

Eligibility criteria were based on the PICO approach. Inclusion was based on the following criteria:

- A study population with a medical diagnosis of glycogen storage disease, adults (⩾18 years).
- Studies comparing the effects of all forms of physical training programmes including aerobic training in the form of swimming, cycling, walking, jogging, flexibility, strength and respiratory muscle training.
- Interventions undertaken for a period of at least 4 weeks to ensure sufficient time for aerobic or respiratory conditioning to occur, but no upper limit on study duration.

Papers were excluded that were unrelated, duplicated, non-human subjects, subject requiring continuous invasive/continuous NIV, unavailable full texts, abstract only papers, editorials, reviews, authors responses and books.

### Search strategy

#### Electronic searches

We searched SCOPUS (1966 to February 2020) using the following search terms: {glycogen storage disease} AND ‘exercise’ OR ‘endurance training’ OR ‘ aerobic exercise’ OR ‘physical fitness’ OR ‘muscle training’ OR ‘resistance training’ OR ‘ aerobic conditioning’ OR ‘respiratory training’ OR ‘walk*’ OR ‘swim*’ OR ‘cycl*’ OR ‘jogging’. Limited to human/humans’ papers.

We searched MEDLINE (1950 to February 2020) using a modified search strategy to include exploded MeSH Headings: (MeSH HEADING:exp: (((((((((((glycogen storage disease) OR Fanconi Syndrome) OR Glycogen Storage Disease Type I) OR Glycogen Storage Disease) OR Glycogen Storage Disease Type II) OR Glycogen Storage Disease Type III) OR Glycogen Storage Disease Type IV) OR Glycogen Storage Disease Type V) OR Glycogen Storage Disease Type VII) OR Glycogen Storage Disease Type VI) OR Glycogen Storage Disease Type VIII) OR Glycogen Storage Disease Type IIb) *AND* MeSH HEADING:exp: ((((((exercise) OR Exercise Test) OR Exercise Therapy) OR Exercise) OR Exercise Tolerance) OR Muscle Stretching Exercises) OR Resistance Training)) *OR* MeSH HEADING:exp: (respiratory training OR Breathing Exercises). All searches were conducted on 11 February 2020.

### Selection of studies

Two authors (P.H., C.B.) independently reviewed abstracts in order to identify potential studies for inclusion. Full texts were downloaded and screened for inclusion according to eligibility criteria by two researchers independently. Any disagreement was resolved by consensus agreement following discussion with another author (I.V.). In addition, the reference list of eligible papers was checked to ensure that all potential eligible papers had been identified. Foreign language studies were translated into English.

### Quality assessment

Publications were assessed for quality by considering characteristics that could introduce bias using the NIH Quality Assessment Tool for Before-After (Pre-Post) Studies with no control group and the NIH Quality Assessment Tool for controlled intervention studies^37^ (Additional file 2).

### Data extraction

Data from the included studies were extracted into defined tables by a single reviewer. Information was recorded on population characteristics, study design, intervention and outcomes. For Aerobic and Strength training, outcomes were categorised into the following groups: Cardiorespiratory fitness; Muscular strength; Functional capacity and Well-being; and Ventilatory function (where data available). For Respiratory Muscle Training, outcomes were categorised into the following groups: Muscular strength; Ventilatory function; and Functional capacity and Well-being. Data presented in graphs were extracted by Web plot digitiser.^38^

## Results

### Search results

**Figure 1.**
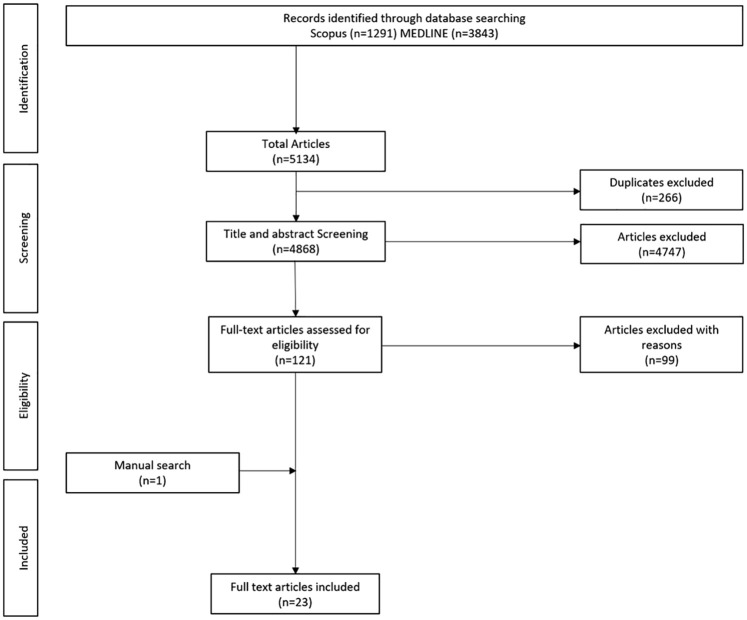
PRISMA flow diagram of literature screening and selection

A total of 4868 titles and abstracts were screened following the search, 121 of which were included for full test screening. Twenty-three articles were subsequently selected for final inclusion in this review (Figure 1). All identified studies included adult patients with McArdle or Pompe disease. Eight studies of adults with McArdle disease were identified, with seven studies investigating aerobic exercise^17,26,27,39–42^ and one study investigating strength training.^28^ Fifteen studies of adults with Pompe disease were identified, with six investigating a combination of aerobic and muscular training;^43–48^ two investigating aerobic and nutrition interventions^49,50^ and seven investigating RMT.^35,51–56^ No studies including adults with other GSDs were identified. The 23 included publications were largely uncontrolled intervention trials. Other publications included single-arm A-B-A experimental design,^53^ a double-blind RCT,^35^ a A-B-C single-arm experimental design,^56^ a longitudinal observation study,^55^ an uncontrolled prospective study^50^ and a quasi-experimental reversal design study.^28^

Outcomes reported in patients undergoing aerobic and muscular training included markers of cardiorespiratory fitness, muscular strength and functional capacity and well-being. Markers of cardiorespiratory fitness included maximum work rates, VO_2_ peak, submaximal VO_2_, ventilatory threshold (VT), gross efficiency (GE), heart rate (HR) and cardiac output. Markers of muscular strength included peak power, Medical Research Council sumscore (MRCss), isometric strength and repetitions of weights. Markers of ventilatory function included vital capacity (VC). Markers of functional capacity included grip strength, timed function tests (TFTs), functional status (Walton & Gardner-Medwin Scale) and muscle function deterioration. All well-being outcomes were self-reported, with validated surveys such as Short Form-36,^39,42,44,49^ Fatigue Severity Scale (FSS)^44^ and Phenotype Severity Scale used.^28^ In patients undergoing RMT, markers of respiratory muscle strength included maximum inspiratory pressure (MIP), maximum expiratory pressure (MEP) and diaphragm thickness. Markers of ventilatory function included peak cough flow (PCF), forced vital capacity (FVC), forced expiratory volume in 1 s (FEV1), MMRC-Dyspnoea and capillary capnometry. Functional capacity and well-being markers included functional status (WGMS), TFTs, QoL using the Nottingham Health profile (NHP), Quality of sleep using the Pittsburgh Sleep Quality Index (PSQI), reported outcomes of fatigue (FSS) and daytime sleepiness (ESS) and Dyspnoea (SRGQ and MMRC Dyspnoea Scale).

### Findings

The following analysis focusses on the effectiveness of exercise in McArdle and Pompe disease. The main exercise interventions identified were aerobic training, muscular training and respiratory muscle training.

#### Aerobic training in McArdle disease

*Characteristics and quality.* Aerobic training involving 34 adults with McArdle disease was included in 7 studies, 3 of which were deemed fair quality^17,26,42^ with the remaining 4 studies being deemed of poor quality.^27,39–41^ The basic characteristics of these are shown in Table 1. All of these were nonrandomised intervention studies, with two studies including controls of clinically healthy subjects; however, these were only included for comparison of acute exercise responses at baseline.^17,27^ The duration of training varied from 4 to 32 weeks, with the frequency of training between 3 and 5 times per week and protocols including 60 min or less of walking and running and cycling, with the majority at an intensity of between 60% and 85% max HR.^17,26,27,39–42^

**Table 1.** McArdle disease: population characteristics and study design.

Author	Quality rating	Population (age)	Study design
Olivier *et al.*^27^	Poor	5 McArdle patients (35 ± 11 years)	Nonrandomised intervention
Haller *et al.*^26^	Fair	8 McArdle patients (33–61 years)	Nonrandomised uncontrolled intervention
Lucia *et al.*^40^	Poor	1 McArdle and Myasthenia gravis patient (29 years)	Nonrandomised uncontrolled intervention
Mate-Munoz *et al.*^17^	Fair	10 McArdle patients (36 ± 5 years)	Nonrandomised intervention
Perez *et al.*^41^	Poor	1 McArdle patient (38 years)	Nonrandomised uncontrolled intervention
Porcelli *et al.*^42^	Fair	7 McArdle patients (41 ± 13 years)	Nonrandomised uncontrolled intervention
Cakir *et al.*^39^	Poor	2 McArdle patients (33 years, 42 years)	Nonrandomised uncontrolled intervention
Santalla *et al.*^28^	Fair	7 McArdle patients (23–58 years)	Quasi experimental reversal

*Outcomes.* Cardiorespiratory fitness: Aerobic capacity was shown to improve with increased VO_2_ Peak observed in five studies (shown in Table 2).^17,26,40–42^ Greatest improvements were found by Perez *et al.*^41^ (14.6 to 30.8 ml/kg/min), Lucia *et al.*^40^ (8.5 to 17.0 ml/kg/min) and Mate-Munoz *et al.*^17^ (13.0 ± 3.8 to 18.8 ± 5.9 ml/kg/min). Smaller differences were seen by Haller *et al.*^26^ (1.3 to 1.5 L/min) and Porcelli *et al.*^42^ (18.5 ± 1.8 to 21.6 ± 1.9 ml/kg/min). In contrast, no differences were found by Olivier *et al.*^27^ VT increased in Lucia *et al.*^40^ (6.1 to 11.8 ml/kg/min) and Mate-Munoz *et al.*^17^ (9.4 ± 1.8 to 12.8 ± 3.7 ml/kg/min). Gross muscle efficiency was only found to increase by Perez *et al.*^41^ (13.8% to 17.2%). Three studies showed improvements in peak heart rate (Perez *et al.*^41^ 140 to 166 bpm; Mate-Munoz *et al.*^17^ 146 ± 22 to 156 ± 19 bpm; Lucia *et al.*^40^ 141 to 163 bpm), whereas others found no differences.^42^ Similar increases were found in cardiac output (Haller *et al.*^26^ 13.1 to 15.0 L/min; Porcelli *et al.*^42^ 15.2 ± 1.3 to 18.9 ± 1.1 L/min). Peak work rate in an incremental test increased in all five studies that measured this^17,26,40–42^ with the highest overall increase being 59 to 120 W.^41^ Significant increases were reported by Haller *et al.*^26^ (59 to 78 W), Mate-Munoz *et al.*^17^ (0.8 ± 0.2 to 1.1 ± 0.3 W/kg) and Porcelli *et al.*^42^ (73 ± 13 to 89 ± 12 W).

**Table 2.** Aerobic and strength training in McArdle disease..

Author	Duration (weeks)	Frequency (days/week)	Protocol of sessions	Outcomes		
				Cardiorespiratory fitness	Muscular strength	Functional capacity and well-being
Aerobic training						
Olivier *et al.*^27^	8	3	Cycling 35–45 min Intensity 60–70% max HR Then 60 min recovery	**Submaximal VO_2_:** No differences **HR**: Decreased at submaximal workloads	–	–
Haller *et al.*^26^	14	4	Cycling 30–40 min Intensity 60–70% max HR	**Peak Work rate** Increased 36% (+19 watts) (*p* < 0.002) **VO_2_ Peak during prolonged test:** Increased 14% (+0.2 L/min) **Peak Cardiac output (Q):** Increased 15% (+2 L/min) (*p* < 0.02)	–	–
Lucia *et al.*^40^	12	5	Walking ⩽60 min Intensity 60% max HR	**Peak Work rate** Increased 61% (+38 watts) **VO_2_ Peak:** Increased 100% (+8.5 ml/kg/min) **VT:** Increased 93% (+5.7 ml/kg/min) **Peak HR:** Increased 16% (+22 bpm)	–	Self-reported improved sense of well-being and ability to perform activities of daily living
Mate-Munoz *et al.*^17^	32	5	Walking and cycling and gentle running ⩽60 min at 60% peak HR	**Peak Work rate:** Increased 38% (+0.3 W/kg) (*p* = 0.014) **VO_2_ Peak:** Increased 45% (+5.8 ml/kg/min) (*p* = 0.006) **VT:** Increased 36% (+3.4 ml/kg/min) (*p* = 0.012) **GE:** No differences (*p* = 0.476) **Peak HR:** Increased 7% (+10 bpm) (*p* = 0.05)	–	–
Perez *et al.*^41^	28	3–4	Running ⩽60 min Intensity ⩽80–85% max HR	**Peak Work rate:** Increased 103% (+61 watts) **VO_2_ Peak:** Increased 111% (+16.2 ml/kg/min) **GE:** Increased 3.4% **Peak HR:** Increased 19% (+26 bpm)	–	Self-reported improvement in well-being and ability to perform activities of daily living
Porcelli *et al.*^42^	12	4	1) 10–15 min Stretching exercises 2) 30–45 min cycling 65–70% max HR	**Peak Work rate:** Increased 22% (+16 watts) (*p* = 0.02) **VO_2_ Peak:** Increased 17% (+3.1 ml/kg/min) (*p* = 0.02) **GE:** Increased 0.8% **Peak HR:** No differences **Peak SV:** Increased 22% (19.9 ml) (*p* < 0.05) **Peak Cardiac Output (Q):** Increased 24% (+3.7 L/min) (*p* = 0.04)	–	**Quality of life:** No differences
Cakir *et al.*^39^	4	5	1) Walking 30–45 min Intensity 5–44% 2) 3 reps of static stretching 3) Diaphragmatic breathing also included	–	–	**Grip strength:** R: Increased 9% (+2.0 kg) L: Increased 14% (+1.8 kg) **10 m walking time:** Decreased 15% (−1.08 s) **Time to climb 4 steps:** Decreased 7% (−0.17 s) **Sit to stand within 30 s:** Increased 14% (+1.5 s). **Quality of life:** Improvement in all scores, except for physical function, vitality, and emotional role in one patient
Strength training						
Santalla *et al.*^28^	16 (+8 detraining)	2	1) Warm up (12 min on arm crank ergometer and 12 min on cycle ergometer) 2) Circuits using large muscle groups, 5–6 reps, using load (kg) eliciting RPE of 6–7. Bench press, leg press, lateral pull down, abdominals 3) Passive stretching (3 × 30 s for each muscle group)	–	**Peak Power:** **Bench press:** Increased 100% (+52 watts) (*p* = 0.018) **Half Squat:** Increased 151% (+173 watts) (*p* = 0.018)	All changed to a lower severity class

GE, gross efficiency; SV, stroke volume; VT, ventilatory threshold; Heart Rate (HR), Rating of perceived exertion (RPE).

Functional capacity and well-being: Improvements in functional capacity (grip strength and 10 m walk) were observed by Cakir *et al.*^39^ (Right grip: 21.5 to 23.5 kg; Left grip: 20.5 to 22.5 kg; 10 m walk: 7.0 to 5.9 s). Improvements in well-being were reported in three studies^39–41^ with two studies documenting self-reported improvements in well-being^40,41^ and others reporting improvements in all QoL scores.^39^

Adherence: In the only study to report this outcome, Porcelli *et al.*^42^ observed 96% adherence to their training programme.

#### Strength training in McArdle disease

*Characteristics and quality.* Strength training was included in one study consisting of seven patients with McArdle disease^28^ (Table 1). This was a quasi-experimental reversal trial deemed of fair quality, consisting of resistance training including a warmup, circuit training of large muscle groups followed by passive stretching, carried out twice per week for 16 weeks.

*Outcomes.* Muscular power was shown to increase after resistance training (Table 2; Bench press: 51 to 103 W; Half Squat: 116 to 290 W).^28^

Adherence: Adherence to training was reported to be between 84% and 100%.^28^

#### Aerobic and strength training in Pompe

*Characteristics and quality.* A combination of aerobic and resistance exercises was investigated in 4 studies consisting of 29 of adults with Pompe disease.^43,44,46,48^ The basic characteristics of these studies are shown in Table 3. These were a mixture of poor,^46^ fair^48^ and good quality studies.^43,44^ Participants were reported to be receiving ERT in three studies^43,44,48^ and hormone replacement therapy in one study.^46^ Two studies included patients supported by walking aids^43,44^ and others included patients supported with NIV.^46^ All of these studies were non-randomised uncontrolled intervention studies, with Van den Berg *et al.*^43^ and Favejee *et al.*^44^ reporting on different outcomes from the same study population. Interventions included aerobic and strength^46,48^ and aerobic, strength and core stability exercise.^43,44^ The frequency of training was 3 times per week with a varied duration between 12 and 20 weeks. Aerobic training consisted of 30 min cycling.^43,44,46,48^ Strength training included major muscle groups, using either body weight or weights^46,48^ or exercise machines.^43,44^ In addition, core stability exercises were included in two studies.^43,44^

**Table 3.** Pompe disease: population characteristics and study design.

Author	Quality rating	Participants with LOPD (age)	Study design
Leutholtz and Ripoll^46^	Poor	*N* = 1 (24 years)	Nonrandomised uncontrolled intervention trial
Terzis *et al.*^48^	Fair	*N* = 5 (36–71 years)	Nonrandomised uncontrolled intervention trial
Van den Berg *et al.*^43^	Good	*N* = 23 patients (46 years; 19.6–70.5)	Nonrandomised uncontrolled intervention trial with staggered starts
Favejee *et al.*^44^	Good	*N* = 23 patients (46 years; 19.6–70.5)	Nonrandomised uncontrolled intervention trial
Sechi *et al.*^49^	Good	*N* = 13 (49 ± 11.0 years)	Partially blinded, randomised, crossover study
Slonim *et al.*^50^	Fair	*N* = 34 (44 ± 11 years)	Uncontrolled prospective study
Montagnese *et al.*^47^	Poor	*N* = 2 (52 and 74 years)	Nonrandomised uncontrolled Intervention trial
Khan *et al.*^45^	Poor	*N* = 1 (34 years)	Nonrandomised uncontrolled intervention trial

LOPD, late-onset Pompe disease.

*Outcomes.* Cardiorespiratory fitness: VO_2_ peak increased after aerobic and strength training and was broadly consistent with aerobic training alone (Table 4; Van den Berg *et al.*^43^ 22.1 ± 7.0 to 24.1 ± 7.1 ml/min/kg). Increases were also found in VT (16.7 ± 4.3 to 18.5 ± 4.7 ml/min/kg) and maximum work rate (110 ± 52 W to 122 ± 53 W).^43^

**Table 4.** Aerobic and muscular interventions in Pompe disease..

Author	Duration (weeks)	Frequency (days/week)	Protocol of sessions	Outcomes			
				Cardiorespiratory fitness	Muscular strength	Ventilatory function	Functional capacity and well-being
Aerobic and strength training							
Leutholtz and Ripoll^46^	12	3	**Aerobic exercise:** 30 min Cycle RPE 11 to 13 of Borg scale 30 min **Strength training:** 30% of 1RM, 12–15 reps using ‘Pyramid’ CAM assisted circuit machines	–	**Repetitions: (at wt. 10 lbs)** **Curls:** Increased 50% (10–15) **Leg ext.:** Increased 42% (7–10) **Pullovers:** Increased 100% (10–20) **Chest press:** Increased 100% (10–20)	**VC:** Increased 31% (1.2–1.5 L)	**Sit to stand:** Unable to complete
Terzis *et al.*^48^	20	3	1) 30 min cycling 2) 10 min stretching of major muscle groups. 3) Resistance exercises of major muscle groups (1–3 sets of 10 reps, resistance 50% 10 reps max.	–	**Knee extension:** (MVC) Increased 46% (+2.3 kg) (*p* > 0.05) **Hip extension:** (MVC) Increased 51% (+2.3 kg) (*p* < 0.05). **Bench press:** (MVC) Increased 25% (+3.0 kg) (*p* < 0.05). **Rowing:** (MVC) Increased 19% (+3.1 kg) (*p* < 0.05)	–	**6MWT:** Increased 22% (+44 m) (*p* < 0.01)
Van den Berg *et al.*^43^	12	3	1) 5 min Warm-up, intensity 100–110 bpm 2) 15 min Cycling (Intensity 60% VO_2_ max) 3) Strength training (Weight 70% of 4 RM, 3 sets 15–20 reps) 4) 15 min Cycling 5) Core stability (3 sets of 30 s) 6) 5 min Cool down	**Peak Work rate:** Increased 11% (+12 watts) (*p* < 0.01) **VO_2_ peak:** Increased 9% (2.0 ml/min/kg) (*p* < 0.01) **VT:** Increased 11% (+1.8 ml/min/kg) (*p* < 0.01)	**Hip flexors:** (MVC) Increased 15% (+24 N) (*p* < 0.01) **Shoulder abductors:** (MVC) Increased 5% (+7.6 N) (*p* = 0.02) **Others:** No differences	**VC:** No differences	**6MWT:** Increased 3% (+16 m) (*p* = 0.01) **Time to climb 4 steps:** Decrease 12% (−0.3 s) (*p* = 0.02) **Rise to standing:** Decrease 17% (−1.0 s) (*p* = 0.05) **Others:** No difference
Favejee *et al.*^44^	12	3	As Van den Berg *et al.*^43^	–	–	–	**Fatigue:** Decreased 10% (Medians: 5.33 to 4.78) (*p* = 0.007) **Pain:** Decreased 35% (*p* = 0.040). **Mental Health:** No differences
Aerobic and strength training plus dietary intervention							
Sechi *et al.*^49^	26	4	**Aerobic exercise:** 1) Warm up 2) 10–15 min Stretching and balance 3) 15 min Strength, moderate loads of main muscle groups using elastic bands. 3 × 10 reps. 4) 30–40 min Cycling (intensity 60% max HR) **Diet:** 25–30% protein, 30–35% carbohydrate, 35–40% fat	**Peak Work rate:** Increased after exercise (medians 75 ± 80 to 75 ± 70 watts (*p* = 0.023) and 16% (+10 watts) after exercise and diet (Medians: 63 ± 55 to 73 ± 55) (*p* = 0.093) **VO_2_ peak:** Increased 10%, (+2.0 ml/min/kg) (Medians: 20.2 ± 7.3 to 22.2 ± 4.6 ml/min/kg (*p* = 0.009) after exercise and diet **VT:** No differences	**Arms extensors, Arm flexors, Leg extensors, Legs flexors:** No differences (MVC)	**VC:** No differences	**6MWT:** No differences **Walton score:** No differences. **General health:** Increased (*p* = 0.03) after exercise and diet **Vitality**: Increased (*p* = 0.03) after exercise and diet **Other components:** No differences
Slonim *et al.*^50^	104–520 (234 ± 130)	7	**Aerobic exercise:** 1) 45–50 min treadmill (or cycle) Intensity 60–65% max HR 2) 10–15 min upper body exercise **Nutrition:** 25–30% protein, 30–35% Carbohydrate 35–40% fat + L-alanine 1.5 g/day 4x/day	–	–	**VC:** No differences	Difference between pre-NET slope of muscle function deterioration to that of post-NET slope was (−0.29 (95% CI, 0.19, 0.39) (*p* < 0.0001)
Vibration training							
Montagnese *et al.*^47^	104	3	2 × 3 min standing 2 × 30 s semi push up position	–	**MRCss**: Increased 14% (+6 N) **Knee extension**: (MVC) Increased 44% (+35.5 bil. Nma) **Arm flexion:** (MVC) Increased 77% (+29.6 bil. Nma)	**VC**: No differences	**6MWT**: Decreased 13% (−19 m) **TFTs and WGMS:** No differences
Khan *et al.*^45^	15	3	Cycle: 60 s vibration on then 60 s vibration off. 2 cycles initially then progressing to 4. Standing	–	**Peak power:** increased 64% (+53 watts). **Knee extensors:** (MVC) Increased 17% (+6 N m) **Flexors:** (MVC) Decreased 13% (−2 N m)	**VC:** No differences	**6MWT:** Increased 70% (+116 m) **Mean grip:** No differences

CI, confidence interval; VC, vital capacity; VT, ventilatory threshold; WGMS, well-being markers included functional status; HR, Heart Rate; RPE, Rating of perceived exertion; MVC, Maximal Voluntary Muscle Contraction; RM, Repetition Maximum; 6MWT, Six Minute Walk Test; CAM, Computer aided manufacturing..

Ventilatory function: VC was only found to increase by Leutholtz and Ripoll^46^ (1.2 to 1.5 L).

Muscular strength: Improvements in muscular strength were shown in three studies^43,46,48^ with increases in maximum isometric strength found by Terzis *et al.*^48^ (Hip extension: 4.6 ± 3.3 kg to 6.9 ± 3.7 kg; Bench press: 12.2 ± 5.3 kg to 15.2 ± 7.8 kg, Rowing: 16.7 ± 9.0 kg to 19.8 ± 5.9 kg) using a load transducer and Van den Berg *et al.*^43^ (Hip flexors: 156.4 ± 61.9 N to 180.7 ± 57.7 N, Shoulder abductors: 143.1 ± 29.1 N to 150.7 ± 35.4 N) using hand-held dynamometry of individual muscle groups. An increase in resistance training repetitions (Curls: 10 to 15; Leg extensions: 7 to 10; Pullovers: 10 to 20; Chest presses: 10 to 20) using a 10-lb weight was observed by Leutholtz and Ripoll.^46^

Functional capacity and well-being: Functional capacity was shown to improve in two studies^43,48^ with improvements found by Terzis *et al.*^48^ (6MWT: 204 ± 177 m to 248 ± 184 m) and Van den Berg *et al.*^43^ (6MWT: 492 ± 89 to 508 ± 97 m; Time to climb four steps −0.54 to −0.04 s and supine-stand time: −2.0 to 0.01 s), in contrast to others.^46^ Well-being outcomes included reduced fatigue (median scores: 5.33 to 4.78) and the numbers of patients reporting pain (13/23 *versus* 5/23).^44^

Adherence: Adherence was only reported in 1 of 3 studies, which found high (89%) rates of session completion.^43^

#### Aerobic, strength and nutrition interventions in Pompe

*Characteristics and quality.* Aerobic, strength and nutrition interventions were described by 2 studies, consisting of 47 adults with LOPD, which were deemed of good^49^ or fair quality^50^ (shown in Table 3). Participants were receiving ERT in the study by Sechi *et al.*^49^ with some patients supported by NIV in the study by Slonim *et al.*^50^ These studies were a partially blinded, randomised crossover study^49^ and an uncontrolled prospective study.^50^ There was a large variation in duration from 26 weeks to 10 years, at a frequency of training between 4 and 7 times per week. Aerobic training consisted of 30–40 min cycling (at 11–13 Borg Scale or 60% VO_2_ or 60% max HR) or 45–50 min (at 60–65% max HR) on a treadmill, with strength training including 10–15 min using exercise machines or resistance bands. All patients were recommended to consume a caloric distribution of 25–30% protein, 30–35% carbohydrate and 35–40% fat, with the ingestion of l-alanine, 1.5 g, 4 times/day by Slonim *et al.*^50^ In contrast, patients were randomly assigned to exercise alone or exercise + diet by Sechi *et al.*^49^ in which the dietary intervention was a personalised high protein diet (25–30% protein, 30–35% carbohydrate, 35–40% fat).

*Outcomes.* Cardiorespiratory fitness: Significant improvements were reported in peak work rate after aerobic exercise alone (although no difference in median was found 75 ± 80 to 75 ± 70 W). With aerobic exercise and diet there were improvements in peak work rate (median 63 ± 55 to 73 ± 55 W) and VO_2_ Peak (median: 22.2 ± 7.3 ml/min/kg to 22.2 ± 4.6 ml/min/kg).^49^

Ventilatory function: VC did not change.^49,50^

Muscular strength: No differences were found by Sechi *et al.*;^49^ however, there was a significant improvement in muscle function deterioration after nutrition, aerobic and resistance exercise by Slonim *et al.*^50^ [−0.29 (95% CI −0.36, −0.19)].

Functional capacity and well-being: Significant improvements in general health and vitality after aerobic, resistance exercise and nutrition were also found.^49^

Adherence: Twenty-six of the thirty-four patients were considered to have moderate to good compliance to the combined exercise and nutrition intervention.^50^ Similarly, Sechi *et al.*^49^ found reasonable adherence to exercise (69% for warm up, 61% for strength training, 74% for cycling, 69% for stretching) with no significant difference between arms. Within the dietary intervention, the percentage of calories introduced with proteins was 96% (median value) of those prescribed and significantly increased from habitual diet.

#### Strength training in Pompe disease

*Characteristics and quality.* Whole body vibration training (WBVT) and side alternating vibration training (SAVT) were described by two nonrandomised uncontrolled intervention studies which were deemed of poor quality^45,47^ and included three adults with LOPD (shown in Table 3). Both studies included patients supported by walking aids, with some receiving ERT and additional regular physiotherapy.^47^ Training was carried out 3 times per week; however, there was a large variation in duration of training ranging from 15 to 104 weeks.^45,47^

*Outcomes.* Improvements in muscular strength included increases in MRCss (41.5 to 47.5), knee extension (70.8 to 106.3 N m), arm flexion (38.3 to 67.9 N m)^47^ and peak power using jumping mechanography (83 to 136 W).^45^ Improvements in functional capacity were also found (6MWT: 166 to 282 m)^45^ (shown in Table 4).

Adherence: This was not reported in either study.

#### Respiratory interventions in Pompe disease

*Characteristics and quality.* Respiratory muscle training was described in a total of 7 studies consisting of 60 adults with LOPD (shown in Table 5).^35,51–56^ Most of the studies were deemed fair quality,^51,53,55,56^ with only Jones *et al.*^35^ deemed fair to good quality.

**Table 5.** Pompe disease: population characteristics and study design.

Author	Quality rating	Participants with LOPD (age)	Study design
Martin *et al.*^54^	Poor	*N* = 1 (42 years)	Nonrandomised uncontrolled intervention trial
Mitja *et al.*^55^	Fair	*N* = 8 (13–58 years)	Longitudinal observational study
Aslan *et al.*^51^	Fair	*N* = 8 (23–64 years)	Nonrandomised uncontrolled intervention trial
Wenninger *et al.*^56^	Fair	*N* = 11 (50 ± 15.6 years)	Prospective monocentric unblinded single-arm pilot study A-B-C design
Jones *et al.*^52^	Poor	*N* = 2 (55 and 64 years)	Nonrandomised uncontrolled intervention trial
Jones *et al.*^53^	Fair	*N* = 8 (49.3 ± 8.4 years)	A-B-A single subject experimental design
Jones *et al.*^35^	Fair to Good	*N* = 22 RMT: 12 patients (53.2 ± 12.7 years) Sham-RMT:10 patients (46.6 ± 13.9 years)	Double-blind randomised control trial

LOPD, late-onset Pompe disease; RMT, respiratory muscle training.

The population characteristics and study design are shown in Table 5. All included patients had respiratory muscle weakness, with five papers including a subset of patients receiving noninvasive nocturnal ventilation (including between 1 and 13 patients).^35,51,53,54,56^ Adjuvant ERT treatment was used in all but one study.^54^ Three studies were nonrandomised uncontrolled trials.^51,52,54^ Jones *et al.*^35^ was the only double-blind RCT. Others included an A-B-A single subject trial,^53^ a single-arm pilot study A-B-C design^56^ and a longitudinal observational study.^55^ A summary of the interventions and outcomes are shown in Table 6. All of the papers included RMT, with both Inspiratory Muscle Training (IMT) and Expiratory muscle training (EMT) used in three studies^35,52,53^ and IMT used alone in four studies.^51,54–56^

**Table 6.** Respiratory muscle training in Pompe disease..

Author	Duration (weeks)	Frequency (days/week)	Intensity (% MIP or MEP)	Protocol of sessions	Outcomes		
					Muscular strength	Ventilatory function	Functional capacity and well-being
Inspiratory muscle training							
Martin *et al.*^54^	15	7	6–46 cmH_2_O L/sec	15 min x 2/day.	**MIP:** Increased 45% (+27.0 cmH2O) **MEP:** Increased 70% (+42.0 cmH2O)	**FEV_1_**: No differences **FVC:** Decreased 7% (−0.32)	
Mitja *et al.*^55^	96	7	30	**Cycle:** 1′ at 30% MIP then 2′ deep slow breathing 15 cycles per day for 45 min (15′ at 30% MIP and 30′ at rest with deep slow breathing)	**MIP:** Increased 18% (+5.6 cmH_2_O) (*p* = 0008) Improvements stable over the course of the study (*p* < 0.05) **MEP:** No differences	**FVC:** No differences	**Gardner-Medwin-Walton scale:** No differences
Aslan *et al.*^51^	8	⩾5	30 initially, increased weekly by 2 cmH_2_O	15 min, twice/ day 80 sessions per patient	**MIP:** Increased 30% (+9.0 cmH_2_O) (*p* = 0.01) **MEP:** No differences	**FVC, FEV1, PCF:** No differences	**Quality of life:** **Social isolation scores:** Improved (Medians: 22.5 (22.1–69.8) to 0.0 (0.0–16.9) (*p* = 0.02) **Other Subscores**: No differences **Sleep Quality:** No differences
Wenninger *et al.*^56^	6 (+6 detraining + 40 optional training period)	5	30–40 initially then optional increase by 10–15.	30 min daily 7 × 15 inhalations each 525 IMT reps per week	**MIP:** Increased 16% (+7.6 cmH_2_O) (*p* = 0.024) Increased 26**% (+13.4 cmH_2_O)*** (*p* = 0.001) **MEP:** No differences	**FVC, FEV1, Capillary capnometry:** No differences	**6MWT, quality of life (SGRQ, MMRC-Dysnea scale):** No differences
Inspiratory and expiratory muscle training							
Jones *et al.*^52^	16–32	6	⩾60	2 × 25 reps of IMT or EMT daily for 4–10 weeks then both IMT and EMT	**MIP:** Increased 65% (+18.0 cmH_2_O) **MEP:** Increased 39% (+17.0 cmH_2_0)	**FVC:** No differences*	
Jones *et al.*^53^	12	5	60–70	3 × 25 reps of IMT and EMT daily **Overall:** 4500 reps IMT 4500 reps EMT	**MIP:** Increased 20% (+8.6 cmH_2_O) **MEP:** Increased 16% (+11.6 cmH_2_O)	**PCF:** Increased 12% (+1.0 L/s)****	**6MWT:** Increased 2.2% (+7 m) **Supine to stand:** Decreased 13% (−1.6 s) **Stair climbing:** Decreased 15% (−0.6 s) **10 m walk:** Decreased 3% (−0.3 s)
Jones *et al.*^35^	12	5	**RMT:** 50–70 Sham-RMT: 15	3 × 25 reps of IMT and EMT daily. **Overall:** 4500 reps IMT 4500 reps EMT	**MIP:** No differences (between groups) **MEP:** No differences (between groups) **Diaphragm Thickness:** No differences	**PCF:** No differences **Polysomnography:** no differences	**Time to climb 4 steps:** Decreased 0.9 s in RMT group. Decreased 0.1 s in Sham-RMT group (*p* = 0.0346) **Daytime sleepiness (ESS):** Decreased 1.2 in RMT group. Increased 1.1 in sham-RMT (*p* = 0.0160) (no raw data available) **Other gross motor function and patient-reported outcomes:** No differences (between groups)

EMT, expiratory muscle training; ESS, daytime sleepiness; FEV1, forced expiratory volume in 1 s; FVC, forced vital capacity; IMT, inspiratory muscle training; MEP, maximum expiratory pressure; MIP, maximum inspiratory pressure; PCF, peak cough flow; RMT, respiratory muscle training; 6MWT, Six Minute Walk Test; SGRQ, St. Georges Respiratory Questionnaire; - Not included; * Obtained 10 weeks after discontinuation of RMT in 1 patient and after approximately 6 months of RMT in the second patient; **After 6 weeks of training; ***Over the total study period of 52 weeks including 6 weeks of training, 6 weeks detraining and a 40 week optional training period; **** Only measured in 5 patients.

The majority of the studies had a training period of 15 weeks or less, with two papers of longer training duration of up to 96 weeks.^52,55^ The interventions used pressure threshold RMT which consisted of multiple repetitions of RMT against fixed resistance (threshold) at either a relatively fixed intensity^35,52,53,55^ or progressive intensities over the duration of the training.^51,54,56^ Patients were prescribed between 30 and 45 min training per day, between 5 and 7 times per week.

*Outcomes.* Respiratory muscle strength: Improvements in markers of muscular strength were found in six of the seven studies after IMT alone or with both IMT and EMT (Table 6). Significant improvements in MIP were observed in three studies, specifically after IMT [Mitja *et al.*^55^ 31.6 ± 17.7 to 37.2 ± 19.3 cmH_2_O; Aslan *et al.*,^51^ median 30 cmH_2_O (21.5–48.0) to 39 cmH_2_O (31.2–56.5); Wenninger *et al.*^56^ 48.6 ± 18.0 to 56.2 ± 19.9 cmH_2_O after initial 6-week training period and 48.6 ± 18.0 to 61.4 ± 28.7 cmH_2_O over the total duration of study including an additional training period]. Improvements in MEP were observed in three studies; however, in contrast, these were predominantly observed after inspiratory and expiratory training combined and values showing statistical significance were not presented.^52–54^

Ventilatory function: Improvements in ventilatory function were only found by Jones *et al.*^53^ with increases in PCF (7.5 ± 0.6 to 8.5 ± 1.4 L/s). No differences were found in other ventilatory markers.

Functional capacity and well-being: Improvements in markers of functional capacity were found after IMT and EMT [Jones *et al.*:^53^ 6MWT: Increased 2.2% (+6.9 m); Supine-stand: Decreased 13.4% (−1.6 s), climb 4 stairs: Decreased 15% (−0.6 s), walk 10 m: Decreased 3.4% (−0.3 s) Jones *et al.*:^35^ Climb 4 steps −0.9 s treatment *versus* −0.1 s control, *p* = 0.03]. Well-being including social isolation scores significantly improved [Aslan *et al.*^51^ Median 22.5 (22.1–69.8) to 0.0 (0.0–16.9)] and daytime sleepiness significantly decreased compared to controls (Jones *et al.*^35^ ESS score −1.2 treatment *versus* +1.1 controls).

Adherence: Adherence was excellent, in which Wenninger *et al.*^56^ found 107% of the training sessions were completed. Similarly, Jones *et al.*^53^ found a mean of 99% prescribed IMT repetitions and 101% of prescribed EMT repetitions were completed and Jones *et al.*^35^ found mean adherence to be 98% in the treatment arm and 97% in the control arm.

## Discussion

The aim of this review was to investigate the effectiveness of exercise training in adults with GSDs. Of the recognised 16 forms of GSDs,^7^ no data were available for 14, with exercise interventions only assessed in adults with McArdle or Pompe disease. In McArdle disease, aerobic exercise in five of the seven studies improved aerobic performance,^17,26,40–42^ with further benefits on functional capacity and well-being found by Lucia *et al.*^40^ and Perez *et al.*^41^ Strength training increased muscular strength and reduced disease severity^28^ (see Table 2) and these improvements were found to be retained in all but one patient after a period of 2 months detraining.^28^ In Pompe disease, the literature shows that a combination of aerobic and strength training improves aerobic capacity, muscular strength, functional capacity and well-being (see Table 4). Furthermore, RMT improved respiratory muscular strength in all of the studies of Pompe disease, with additional benefits in aerobic capacity, functional capacity and well-being shown by some (see Table 6). In the current literature, exercise training appears to be safe and beneficial to health in adults with McArdle and Pompe disease and thus there appears to be a growing body of evidence which suggests that supervised exercise training is safe and effective in improving aerobic capacity and muscle function in adults with McArdle or Pompe disease. The literature base is limited, however, in both quality and quantity with a dearth of literature regarding exercise training in other GSD subtypes. Further research of robust design would be beneficial across the spectrum of GSDs.

### Aerobic and strength training in McArdle disease

McArdle disease (OMIM #232600, EC 2.4.1.1) is caused by a deficiency of myophosphorylase (PYGM), which impairs glycogenolysis specifically in skeletal muscle.^5,28,57^ This block in glycogenolysis limits glucose availability (via glycogen) and places greater reliance on blood glucose and non-esterified fatty acids to meet the metabolic needs of skeletal muscle. During exercise, when the metabolic rate is elevated, the impairment in substrate availability can result in an acute energy crisis and result in pain, fatigue and contractures.^15,57^ In acute exercise of moderate intensity, these symptoms can subside as exercise duration persists beyond 10 min due to greater uptake of blood-borne glucose derived from the liver and fat oxidation in contracting muscles.^28,57–59^ However, despite some improvement in exercise capacity during moderate intensity, during longer duration exercise people with McArdle disease still exhibit impaired exercise performance and aerobic capacity compared to a healthy population.^15,28,57^ Due to this, the majority of studies in McArdle disease investigated the effect of aerobic training, which is hypothesised to increase exercise tolerance primarily through an improvement in aerobic capacity that is driven by a training-induced improvement in free fatty acid oxidation (the ‘second wind’ effect). In addition, aerobic training may also counteract the effects of a sedentary lifestyle, which can include an increased dependence of glycogen as a fuel, skeletal muscle atrophy and weakness, and poor cardiovascular fitness.^15^

The current literature supports the hypothesis that aerobic training improves exercise capacity in McArdle disease and provides evidence for the mechanisms involved in the improvement. Exercise capacity improved after aerobic training in adults with McArdle disease with increases in peak work rates reported in all studies that measured this outcome (*n* = 5).^17,26,40–42^ The magnitude of exercise performance gains was not only consistent but appeared substantial, with intervention studies reporting mean improvements in maximal cycling work rates ranging between 22% and 38%^17,26,42^ while case studies reported improvements between 61% and 103%.^40,41^ Although the improvements documented above are promising, the limited study quality hinders our ability to form strong conclusions and the effect of exercise training on exercise capacity is yet to be fully elucidated. It is likely that the greater exercise capacity following training is partly attributable to improvements in oxidative metabolism, with studies finding concurrent increases in VO_2_ peak, indicative of an increase in cardiac output^17,26,40–42^ and increased VT.^40^ Improvements were even found in an individual with severe clinical features and the presence of two neuromuscular diseases.^40^ The magnitude of this training-induced change is in line with those found in a healthy population, indicating a similar relative capability to improve exercise performance and aerobic capacity, albeit from lower baseline values.^60^

The literature indicates that exercise training in McArdle disease elicits physiological adaptations at several steps of the oxygen cascade, which underpin the improvement in exercise and aerobic capacity, most notably improved cardiovascular function, and muscle oxidative metabolism. Cardiovascular function was markedly improved by training, with 15–24% increases in peak cardiac output^26,42^ achieved via improvements in both peak HR^17,40,41^ and peak stroke volume (SV).^42^ Furthermore, lower heart rates were recorded during submaximal exercise, also indicating improved SV.^27,42^ Although the changes in cardiovascular function are considerable and certainly beneficial to overall health,^61^ they are likely to facilitate the training-induced improvements in aerobic and exercise capacity, rather than drive them. The primary training adaptation in McArdle disease patients is most likely related to improvements in skeletal muscle oxidative metabolism. In the most in-depth study, Porcelli *et al.*^42^ found that 12 weeks of cycling training at 65–70% max HR resulted in a reduction in the O_2_ cost of submaximal exercise (15.8 ± 1.3 to 13.6 ± 1.2 ml/min/W, *p* = 0.03), faster pulmonary VO_2_ kinetics in those with slow VO_2_ kinetics before training and lower submaximal RER. These results indicate that skeletal oxidative metabolism is more efficient following training and that substrate utilisation during submaximal exercise is shifted towards greater free fatty acid utilisation. This is further supported by evidence of an increase in the mitochondrial enzymes citrate synthase and B-hydroxyacyl coenzyme A dehydrogenase.^17,26^ However, these metabolic adaptations to exercise training were not uniform across studies, with Olivier *et al.*^27^ reporting no difference in sub max VO_2_ which is likely attributable to less total training sessions (24 sessions *versus* ⩾60 sessions by others) and Mate-Munoz *et al.*^17^ reporting no differences in GE, potentially due to exercising at a lower intensity compared to others (Table 2). Due to the improvements in cardiorespiratory fitness shown, it is unsurprising that there were improvements in functional capacity^34^ and the ability to perform activities of daily living^40,41^ and QoL.^39^ Surprisingly, despite improvements in outcomes of cardiorespiratory fitness (Peak work rate, VO_2_ Peak, GE, Peak SV and Peak cardiac output), Porcelli *et al.*^42^ found no improvements in habitual levels of physical activity or QoL. However, the post training measurements of physical activity and QoL were taken up to 3 months after the termination of training, thus if any improvements were made, they were not found to be sustained.

Strength training improved strength due, in part, to increases in total and lower body lean mass; these improvements appeared to reduce disease severity. However, these results are from just one small study, thus limiting the validity of findings to the wider population.^28^ Overall, based on the literature reviewed, exercise may be an effective method of reducing symptoms in McArdle disease through improvements in aerobic and physical capacity.

### Aerobic and strength training in Pompe disease

Pompe disease (OMIM #232300, EC 3.2.1.20) is a GSD that also affects skeletal muscle; however, in contrast to McArdle disease, the heterozygous mutation in the GAA gene is within the lysosomes and lysosomal glycogenolysis is blocked. As there is minimal contribution from lysosomal glycogen breakdown to ATP production, it is therefore not a deficiency in ATP resynthesis.^18^ Instead, the deficiency of alpha-1,4-glucosidase causes an accumulation of lysosomal glycogen in skeletal, respiratory and cardiac muscle which leads to exercise intolerance and skeletal muscle weakness and wasting.^5^ A sedentary lifestyle further impacts exercise intolerance, with immobility causing skeletal muscle atrophy, weakness with a low VO_2_ and increased dependence of glycogen as fuel.^15^ As Pompe disease primarily impacts muscle weakness, wasting and physical activity, all studies in adults with Pompe disease focussed on muscular interventions alone or in combination with aerobic training.

The literature shows that a combination of aerobic and strength training increased muscular strength, with improvements observed in three of the four studies which measured this.^43,46,48^ The studies that employed formal weight training protocols with clearly defined levels of resistance were able to increase strength by 5–100% (Table 4); however, these were nonrandomised uncontrolled studies including 23 patients or less who exercised between 12 and 20 weeks.^43,46,48^ The trial which showed no improvement in strength employed resistance band training and a subjective marker of intensity, which may account for the lack of improvement.^49^ Following improvements in muscle strength, subsequent increases in functional capacity were found, with increases in 6MWT,^43,48^ time to climb 4 steps and rise to standing.^43^ The mechanisms underpinning the strength gains were not investigated in depth; however, increased lean body mass (LBM) accompanied strength gains,^48^ indicating participants may have undergone training-induced muscle hypertrophy. Indeed, the magnitude of the change in LBM found by Terzis *et al.*^48^ (+8.4%) is similar to that observed in a healthy population undergoing a similar strength training programme (+8.5%).^62^ These results are encouraging, particularly as the quality of evidence appears greater compared to that of McArdle disease due to the inclusion of large sample sizes and inferential statistics.

Despite only two studies reporting on aerobic performance, significant improvements in maximum work rate,^43,49^ VO_2_ peak and VT^43^ were found indicating that exercise training in Pompe disease elicits physiological adaptations along the oxygen cascade as observed in McArdle disease. Furthermore, the addition of a high protein diet appeared to elicit greater improvements in maximum work rate and an increase VO_2_ peak.^49^ Further to these improvements, reductions in fatigue and pain were also found.^44^ Where aerobic, resistance exercise and diet were combined in an intervention lasting 2–10 years, a reduction in muscle function deterioration was observed, most likely being due to a reduction of glycogen and a reduction in proteolysis, autophagy and muscle damage.^50,63^ Furthermore, Sechi *et al.*^49^ found improvements in general health and vitality, which is unsurprising following the improvements in aerobic capacity.

Vibration training was shown to improve muscular strength,^45,47^ which could be due to muscle adaptations including type two myofiber hypertrophy and an increased muscle cross-sectional area^64,65^ and may explain improvements in 6MWT by Khan *et al.*^45^ Vibration training could therefore offer a time efficient and easily adopted mode of exercise;^45,47^ however, as results were derived from poor-quality case reports,^45,47^ larger studies of enhanced quality are necessary before recommendations can be made.

In summary, all training modalities appear to benefit muscular strength and functional capacity, with improvements in aerobic performance where aerobic exercise is included.

### Respiratory interventions in Pompe disease

Progressive respiratory weakness is prevalent in LOPD^56^ with symptoms such as nocturnal hypoventilation, diaphragm weakness or sleep apnoea becoming apparent before other muscle weakness.^66–68^ Despite ERT, respiratory weakness persists in approximately one third of patients, in which respiratory muscle weakness can decline by approximately 3.2% MIP per year.^69,70^ This leads to reduced airway clearance,^71^ sleep disordered breathing and the requirement of ventilatory support towards the latter stages. In 70% of patients, this can even progress to premature death.^72^

RMT was conducted in adults with LOPD as it offers a therapeutic option using pressure threshold respiratory trainers calibrated to provide inspiratory or expiratory resistance for forced voluntary inspiration/expiration muscle contractions.^52^ As inspiratory muscles are similar to skeletal muscle, they should respond to training and enhance ventilation with increased coordination, endurance and strength.^56^ IMT protocols were therefore conducted in all studies, aiming to target specific inspiratory muscle weakness.^73^

The literature shows that IMT increased inspiratory muscle strength, with MIP improving in six of seven studies.^51–56^ Improvements were observed after just 6 weeks (+15.7% MIP),^56^ increased over the duration of training^52^ and improvements were found to be maintained following training cessation.^35,53,56^ The addition of EMT to IMT also led to increased expiratory muscle strength in the form of MEP.^52,53^ In the only randomised double-blind controlled trial by Jones *et al.*,^35^ despite the magnitude of improvements in MIP and MEP being similar to others,^52,53^ no statistical differences were found between RMT and Sham-RMT arms due to the control arm appearing to elicit an active response. This study was also underpowered and despite randomisation there were differences in baseline characteristics between groups, with those assigned the treatment being older, on ERT for longer, and with increased respiratory muscle involvement. Interestingly, it appears EMT is necessary to elicit improvements in functional capacity^35,53^ which may be due to the role of respiratory muscles in aiding truncal mobility and stabilisation.^73–75^ The evidence appears to be of fair to good quality due to well-defined participant selection, repeat outcome measures and the inclusion of statistics.

Isolating the effect of RMT on the outcomes reported is difficult as all studies included patients receiving ERT which is known to stabilise or even reduce respiratory decline.^76^ However, improvements from ERT largely occur 12–26 weeks after treatment and are usually modest (3–4% for MIP and MEP).^23,76^ Patients had been receiving ERT for at least 10 months in the majority of studies prior to RMT; thus, the improvements shown were likely attributable to RMT alone.^35,52,53,55,56^

#### Safety and adherence

Aerobic exercise in McArdle disease was shown to be well tolerated in the two studies that reported this,^26,27^ with no adverse effects such as muscle injury, contractures, rhabdomyolysis or myoglobinuria. Furthermore, aerobic exercise appeared safe in five studies in which the muscle damage marker creatine kinase (CK) remained stable^26,27^ or even decreased throughout the trial.^17,40,41^ Adherence was only measured in one of the seven studies but was reported to be very good with 96% of training sessions completed, which was most likely due to the high levels of support provided, with weekly phone calls and encouragement given.^42^ Similarly, strength training in McArdle disease was well tolerated, with no adverse effects reported and CK remaining stable. In addition, patients were very complaint, with 100% of sessions completed in five of the seven participants.^28^

Aerobic and strength training in Pompe disease was reported to be well tolerated in the only study that reported on this, with no adverse events observed^48^ and safe with CK levels shown to decrease over the duration of the trial in the only other study that reported on this.^43^ Adherence was reported in four of the six studies;^43,44,49,50^ however, only two studies provided data.^49,50^ Sechi *et al.*^49^ found adherence to exercise was good (61–74%) and Slonim *et al.*^50^ found 26 out of 34 patients were consistently compliant with the nutrition and exercise protocol. Similarly, both studies, including vibration training in Pompe, found it to be well tolerated, with no adverse events reported and CK remaining stable. However, no measures of adherence were reported.^45,47^

Respiratory interventions for Pompe were well tolerated with no adverse effects reported in three of the seven studies that reported this.^51,53,56^ Where side effects were reported, the majority were mild and almost half were unrelated.^35^ Adherence was reported by three of the seven studies and of these it was reported to be excellent, with 107%,^56^ 99% (IMT sessions) and 101% (EMT sessions)^53^ and 98% (treatment arm) and 97% (control arm) completion rates.^35^ Wenninger *et al.*^56^ found adherence to decrease over the full duration of the study, with a drop of 13% (107 to 94%) training sessions completed over the total duration which included 6 weeks of detraining and then a 40-week optional training period.

Overall, aerobic, strength and respiratory muscle training appears to be safe and well tolerated with good adherence in McArdle and Pompe patients. However, this was only reported on in a limited number of studies and should be important factors to include in future studies.

#### Strengths and limitations

The major strength of this review is it is the first to systematically investigate the effectiveness of exercise training across the full spectrum of GSDs, using detailed and reproducible methodology with strict inclusion and exclusion criteria. Considering the full spectrum of GSDs allowed us to identify that exercise training interventions have only been studied in McArdle and Pompe disease, leaving 14 of the 16 GSD types unstudied. We also considered the effectiveness of several exercise training modalities, allowing us to consider the relative benefits of each for both GSD types.

The major limitation of this review was a lack of research into other GSDs, particularly others with skeletal muscle involvement (GSDVII, IXd, X) and those with skeletal muscle and liver involvement (GSD III, GSDXIV), in which the effects of exercise on the skeletal and hepatic presentation could have been investigated.^5,19,77^ Furthermore, the potential impact of exercise on overall general health, fitness and QoL could have been investigated in non-muscular forms of GSDs (GSD0a, GSDIa, GSDIb, GSDVI, GSDIXA1, GSDIXA2, GSDIXc).^30,31^ The majority of the studies included were deemed poor to fair quality, largely due to being uncontrolled, of small sample sizes including several case studies and of short duration. This deficit in study quality impacts interpretation; however, some limitations are to be expected given the rarity of these diseases and the impact this has on recruitment. Regarding study design, it is important to acknowledge that there could have been a learning effect of the pre and post intervention tasks which may have impacted on the results reported. In addition, interpretation of the literature was also impaired by the heterogeneity of training interventions, which varied substantially in regard to modality, duration, intensity and the inclusion of other treatment strategies (e.g. ERT). In those receiving ERT, they had been on this for at least 1 year; therefore, the initial benefits of ERT would have stabilised and thus improvements observed were likely due to the interventions alone.^43,44,47,49^ Furthermore, staggered starts of studies showed patients remained relatively stable and exercise training and nutrition still reduced muscle deterioration in those not receiving ERT.^50^

#### Further research

Further RCTs and intervention studies should include adults with other GSDs particularly those with skeletal muscle involvement as part of larger multicentre randomised studies to clarify the effectiveness, safety and adherence of exercise training across the broad spectrum of GSDs. However, it is acknowledged this would be difficult given the rarity of these diseases. Comparison between individual exercise components within studies would offer greater insights into the most effective methods of training, with further research into vibration training and RMT warranted given the success in Pompe. Further studies of longer duration with multiple follow-up periods would allow us to see if beneficial effects would be maintained long term and if exercise training has wider implications on preventing chronic diseases such as diabetes and cardiorespiratory disease.^27^

## Conclusion

In conclusion, the current evidence shows exercise training appears to be safe and effective in adults with McArdle disease or LOPD, with improvements observed in aerobic capacity, muscular strength and functional capacity. The effect of RMT in Pompe, where sufficiently intense, was also found to be beneficial, with these improvements appearing to be maintained several months after training stopped. However, these findings are largely based on the limited quality of evidence available, largely derived from small uncontrolled intervention studies of short duration that included highly varied exercise protocols which limits the generalisation of findings. Further research of increased quality is required, particularly focussing on how exercise may benefit the clinical course of the disease across the broad spectrum of GSD types.

## Supplemental Material

sj-docx-1-trd-10.1177_26330040221076497 – Supplemental material for A Systematic Review investigating the Effectiveness of Exercise training in Glycogen Storage Diseases Click here for additional data file. Supplemental material, sj-docx-1-trd-10.1177_26330040221076497 for A Systematic Review investigating the Effectiveness of Exercise training in Glycogen Storage Diseases by Claire Bordoli, Elaine Murphy, Ian Varley, Graham Sharpe and Philip Hennis in Therapeutic Advances in Rare Disease

sj-xlsx-1-trd-10.1177_26330040221076497 – Supplemental material for A Systematic Review investigating the Effectiveness of Exercise training in Glycogen Storage Diseases Click here for additional data file. Supplemental material, sj-xlsx-1-trd-10.1177_26330040221076497 for A Systematic Review investigating the Effectiveness of Exercise training in Glycogen Storage Diseases by Claire Bordoli, Elaine Murphy, Ian Varley, Graham Sharpe and Philip Hennis in Therapeutic Advances in Rare Disease
